# Polymyxin heteroresistance in Klebsiella oxytoca

**DOI:** 10.1099/jmm.0.002148

**Published:** 2026-03-27

**Authors:** Eunice A. Ayerakwa, Edward J.A. Douglas, Gerald Larrouy-Maumus, Andrew Michael Edwards, Abiola Isawumi

**Affiliations:** 1 Department of Biochemistry, West African Centre for Cell Biology of Infectious Pathogens, Cell and Molecular Biology, University of Ghana, Accra, Ghana; 2 Department of Biochemistry, Cell and Molecular Biology, University of Ghana, Accra 00233, Ghana; 3 Centre for Bacterial Resistance Biology, Imperial College London, London SW7 2AY, UK; 4 Department of Infectious Disease, Imperial College London, London SW7 2AY, UK; 5 Department of Life Sciences, Imperial College London, London SW7 2AY, UK

**Keywords:** heteroresistance, *Klebsiella*, *Oxytoca*, polymyxin, resistance, serum

## Abstract

**Introduction.** Antibiotic heteroresistance presents a growing public health concern, as the phenotype is associated with treatment failure and is hard to detect using conventional diagnostic testing.

**Gap Statement.** Heteroresistance in *Klebsiella oxytoca*, an opportunistic pathogen associated with hospital-acquired infections, has not been characterized.

**Aim.** In this study, we characterized polymyxin B heteroresistance in a collection of six clinical and environmental isolates of *K. oxytoca*.

**Methodology.** We assessed heteroresistance using population analysis profile assays and LPS modifications using matrix-assisted laser desorption/ionization-time of flight (MALDI-TOF).

**Results.** All six isolates tested exhibited heteroresistance, indicated by an 8–16-fold difference between the MIC of the bulk population and the MIC of the resistant sub-population, as determined using population analysis profiling. Heteroresistance was found to be due to the presence of a stable sub-population of resistant bacteria, the size of which was unaffected by growth phase or the presence of host antimicrobial factors present in human serum. MALDI-TOF analysis revealed 4-amino-l-arabinose modifications of the lipid A of resistant sub-populations.

**Conclusion.** This pilot study identifies that polymyxin heteroresistance in *K. oxytoca* may complicate the treatment of infections caused by this organism.

## Introduction

Carbapenem- and third-generation cephalosporin-resistant pathogens, including *Klebsiella pneumoniae* and *Escherichia coli*, have been classified as a critical priority group by the World Health Organisation (WHO)[1] . Furthermore, other species of *Enterobacterales* are emerging as public health threats due to their ability to cause severe infections and resist multiple antibiotics. *Klebsiella oxytoca* is the second most common cause of *Klebsiella* infection after *K. pneumoniae* [2], with some strains producing extended-spectrum beta-lactamase (ESBL), carbapenemase, imipenemase and/or oxacillinase enzymes [3,5]. Additional resistance mechanisms include efflux pumps that confer resistance to β-lactams and quinolones, respectively [5]. Moreover, several *K. oxytoca* isolates have acquired resistance to colistin, aminoglycosides, fosfomycin, sulphonamides, trimethoprim and macrolides [5]. In keeping with similar pathogens, multiple drug resistance is often mediated by the presence of plasmids that encode diverse Antimicrobial Resistance (AMR) determinants [6].

In humans, *K. oxytoca* is routinely found in the respiratory and gastrointestinal tracts as part of the normal flora [5]. However, the pathogen can establish mild to life-threatening infections, including pneumonia, Urinary Tract Infections (UTIs), bacteraemia, meningitis and antibiotic-associated haemorrhagic colitis in immunocompromised individuals [7,8]. Additionally, *K. oxytoca* has been associated with skin and soft tissue infections such as surgical wounds and necrotizing fasciitis in patients with malignancies and abscesses [5]. In clinical settings, drug-resistant *K. oxytoca* has been responsible for infection outbreaks, most likely due to its ability to survive on fomites and hospital infrastructure such as water reservoirs, ventilators, sinks and disinfectants [9]. The emergence and dissemination of these resistant isolates have restricted treatment options, especially in resource-limited settings, necessitating the use of second- or third-line options such as polymyxins B and E (colistin).

Polymyxins are cationic lipopeptide antibiotics used as a last resort for treating infections caused by multidrug-resistant Gram-negative bacteria [10 [11]]. These antibiotics target LPS, disrupting both the outer and inner membranes, leading to bacterial death [12,13].

Resistance to polymyxins is a growing concern and can arise spontaneously or via the acquisition of a mobile colistin resistance (*mcr*) gene. In both cases, resistance is conferred by modification of the lipid A component of LPS [14].

There is also a growing awareness of polymyxin heteroresistance [10,15], which describes a population-wide variation in antibiotic response among bacterial sub-populations. Generally, two forms of antibiotic heteroresistance have been described; inducible and classical heteroresistance. Inducible heteroresistance may be triggered by antibiotic exposure such that transient resistance occurs in the absence of genetic changes [16]. Conversely, classical heteroresistance describes a sub-population of often highly resistant bacteria, usually due to mutations or gene amplifications, which can be unstable or stable [17].

Polymyxin heteroresistance has been detected in several different members of the *Enterobacterales* and has been implicated in persistent infection and antibiotic therapeutic failure [18,21]. This therapeutic challenge is exacerbated by the difficulties associated with detecting heteroresistance using standard antibiotic susceptibility testing [22]. The current gold standard for heteroresistance detection is population analysis profiling (PAP), which involves quantifying the ability of a bacterial population to grow across a gradient of antibiotic concentrations [21]. The population is considered heteroresistant if the inhibitory concentration for the resistant sub-population is at least eightfold higher than the MIC of the bulk population [23]. However, this approach is time- and resource-demanding and is not typically used in diagnostic laboratories [18,20, 21]. To date, polymyxin heteroresistance has not been reported for *K. oxytoca*.

Given the growing importance of polymyxin antibiotics for treating infections in low-income settings and the challenges associated with diagnostics, the emergence of polymyxin heteroresistance in clinical isolates of *K. oxytoca* [15] would signify a major public health threat. Therefore, the aim of this pilot study was to identify and characterize polymyxin susceptibility in a panel of six clinical and environmental *K. oxytoca* isolates from Ghana.

## Methods

### *K. oxytoca* isolates and growth conditions

Six *K. oxytoca* isolates from a Ghanaian hospital Intensive Care Unit (ICU) were sourced from the ABiola ISAwumi (ABISA) strain collection at the West African Centre for Cell Biology of Infectious Pathogens, University of Ghana. This included three clinical isolates (*Kleb401*, *Blood4a*, *Nasal2a*) from two female patients (5 and 28 years old) and one male patient (46 years old) with bacteraemia who spent >2 weeks in intensive care. Patients had previously been exposed to antibiotics, including co-amoxiclav, levofloxacin and chloramphenicol. No outcome data were available. We also examined three environmental isolates (*ACN*, *Kleb405*, *CT04*) from ICU fomites (door handles and faucets). The isolates were selected based on their high levels of resistance to conventional and last-resort antibiotics, including carbapenems. All isolates were grown in/on cation-adjusted Mueller Hinton broth 2 (±1.5% agar) (Millipore, 90922) at 37 °C with shaking at 180 r.p.m.

### Antimicrobial susceptibility profiling

The susceptibility profile of the strains to commonly used and last-resort antibiotics in healthcare settings (ceftazidime, levofloxacin, chloramphenicol, gentamicin, imipenem, meropenem, colistin, trimethoprim and polymyxin B) was determined using the broth microdilution method [5]. Briefly, the antibiotics were serially diluted twofold across a 96-well plate, with concentrations ranging from 64 to 0.125 µg ml^−1^. One hundred microlitres of overnight cultures, standardized to provide 5×10^5^ c.f.u. per well, was added to each well containing 100 µl of the antibiotics and incubated at 37 °C for 24 h. The MIC of the antibiotics was determined by absorbance measurement at 600 nm using a microplate reader (TECAN, Infinite 200 Pro). The MIC was defined as the concentration of antibiotic that inhibits bacterial growth by >90% (no visible growth by eye).

### PAP

PAP assays were performed using log- or stationary-phase cultures following a previously described protocol [23]. Briefly, a single colony of each isolate was inoculated into 3 ml of Mueller Hinton Broth (MHB) and incubated overnight at 37 °C with shaking (180 r.p.m.). For some assays, human serum was included at 50% of the culture volume to understand if this host-mimicking condition influenced the heteroresistance profile. Bacteria were then used for PAP analysis (stationary phase) or, for log-phase cultures, bacteria were sub-cultured and grown for 3 h with shaking. Seven serial dilutions (10^−1^ to 10^−7^) were prepared from the cultures using PBS as the diluent. Ten microlitres of each dilution was then spread on Mueller Hinton (MH) agar plates containing polymyxin B at a range of different concentrations (128, 64, 32, 16, 8, 4, 2 or 1 µg ml^−1^). The plates were incubated at 37 °C for 24 h. The c.f.u. on each MH agar plate was then used to determine c.f.u. per millilitre by taking into account the dilution factor. Heteroresistance was identified if the highest inhibitory concentration at which there was no growth was ≥8-fold higher than the highest non-inhibitory concentration (the concentration at which ≥80% of the bacterial population grew compared with the inoculum).

### Serum susceptibility assays

Isolates (5×10^5^ c.f.u. ml^−1^) were incubated at 37 °C for 24 h in serial twofold dilutions of human male Blood type type AB serum, which had not been heat inactivated (Sigma, H4522) (from 50% to 0.78% diluted in MHB) to determine their serum resistance profile. Bacterial growth was determined by measuring absorbance at 600 nm with a microplate reader (TECAN, Infinite 200 Pro), with values corrected against the relevant concentration of serum/MHB without bacteria.

### Time-kill assay in human serum

Serum time-kill assays were based on those used previously to measure antibiotic tolerance [24], performed by inoculating 1 ml of human serum (which had not been heat inactivated) with bacteria to 5×10^8^ c.f.u. ml^−1^ and incubated at 37 °C with shaking. Aliquots of the cultures were taken at specific time points (0, 30, 60, 90 and 120 min), serially diluted (10^1^ to 10^7^) in PBS and plated on MH agar. The plates were incubated at 37 °C for 24 h, and the number of c.f.u. was determined as described above.

### Lipid A analysis

Lipid A analysis was performed using the optimized MALDI-TOF MS-based MALDIxin test as described by Dortet *et al*. [25]. Briefly, 50 µl of bacterial culture was centrifuged, and pellets were washed three times in ultra-pure water. The pellets were subjected to mild-acid hydrolysis by resuspension in 2% acetic acid and heating for 30 min at 100 °C. The acid-treated cells were recovered by centrifugation, and the pellets were washed twice and suspended in 50 µl of ultra-pure water. The bacterial suspension (0.5 µl) was loaded onto the target and mixed with 0.5 µl of a super 2,5-dihydroxybenzoic acid matrix. The mixture was air-dried, followed by matrix-assisted laser desorption/ionization-time of flight analysis on a 4800 Proteomics Analyzer (Applied Biosystems, Foster City, CA, USA).

## Results

### Antimicrobial susceptibility profile of *K. oxytoca* isolates

**Fig. 1.**
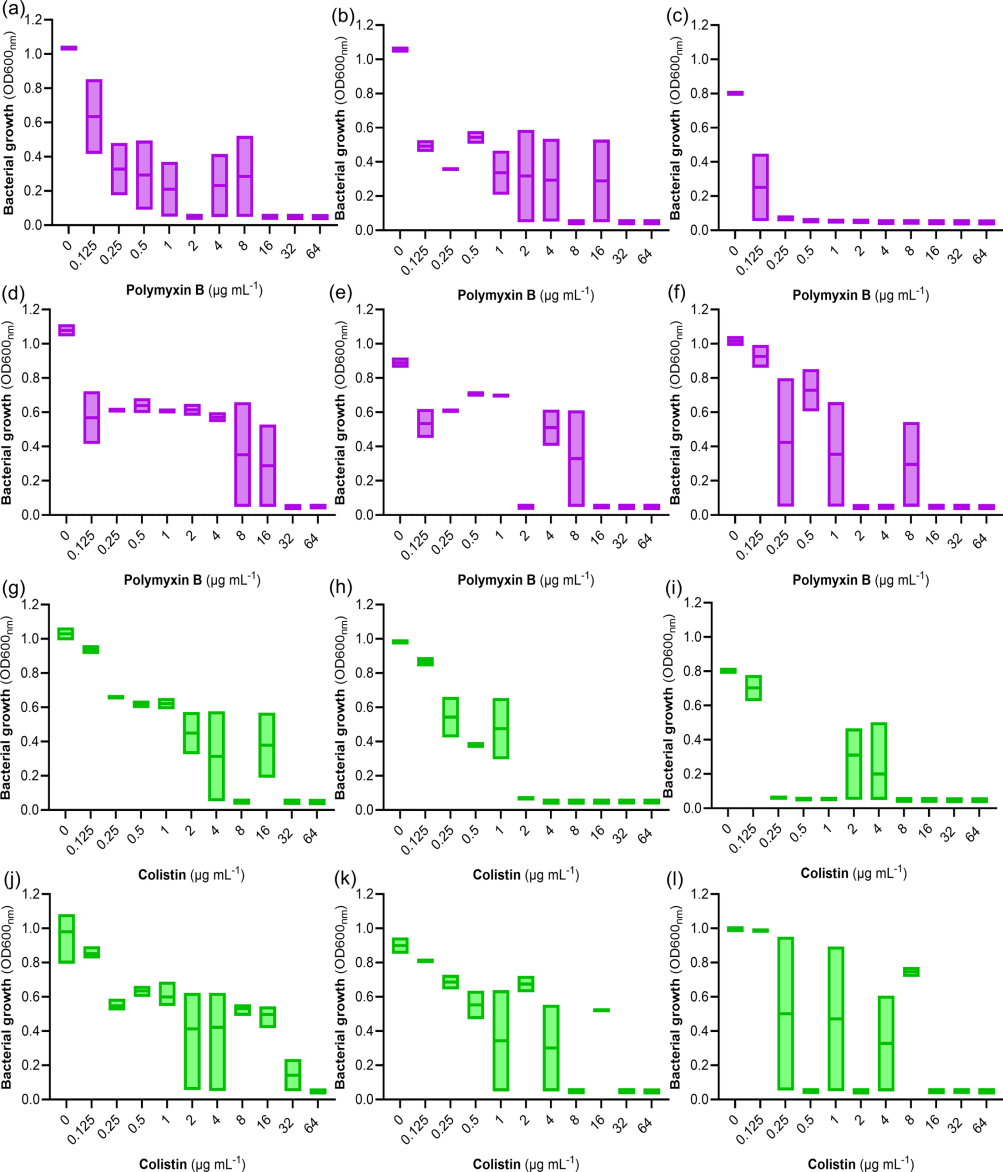
Growth (OD_600_) of strains in the presence of various concentrations of polymyxin B [(a) *CT04*, (b) *Nasal2a*, (c) *Kleb405*, (d) *Blood4a*, (e) *ACN* and (f) *Kleb401*], or colistin [(g) *CT04*, (h) *Nasal2a,* (i) *Kleb405*, (j) *Blood4a*, (k) *ACN* and (l) *Kleb401*]. The data represent three independent broth microdilution assays in MHB. Bars represent the range of OD_600_ values acquired across independent assays for each antibiotic concentration tested. Growth was measured via optiical density measurements at 600 nm wavelength (OD600nm).

To characterize the antibiotic susceptibility of the isolates, we undertook broth microdilution assays to determine the MICs for a range of relevant antibiotics. All six isolates (*CT04*, *Nasal2a*, *kleb405*, *Blood4a*, *ACN*, *kleb401*) were susceptible to ceftazidime, gentamicin and meropenem, with MICs ranging from 0.125 to 2 µg ml^−1^ (Table S1, available in the online Supplementary Material). The isolates were resistant to levofloxacin (MIC ≥32 µg ml^−1^), chloramphenicol (MIC >64 µg ml^−1^), imipenem (MIC ≥16 µg ml^−1^) and trimethoprim (MIC >64 µg ml^−1^) (Table S1). The MICs of colistin and polymyxin B for all six isolates varied between assay replicates and between the two antibiotics, producing a skipped-well phenotype suggestive of heteroresistance [21] (Fig. 1). Skipped wells make MIC determination extremely difficult, which, in turn, makes it challenging to determine whether bacteria are susceptible or resistant. However, polymyxin B at ≤2 µg ml^−1^ inhibited growth of strains CT04, Kleb405, ACN and Kleb401, and colistin at ≤2 µg ml^−1^ inhibited the growth of Nasal2a, Kleb405 and Kleb401 in at least one of the MIC assay replicates (Fig. 1, Table S1). This indicates that the bulk population of most of the strains is likely to be susceptible based on the European Committee on Antimicrobial Susceptibility Testing (EUCAST) breakpoint guidance for polymyxins (26); only strain *Blood4a* was considered resistant to both polymyxin B and colistin based on these assays (Fig. 1).

### Phenotypic characterization of polymyxin B heteroresistance in *K. oxytoca*

**Fig. 2.**
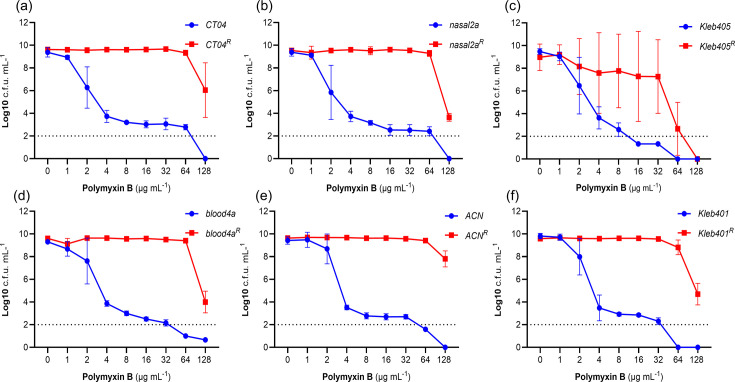
Polymyxin heteroresistance in *K. oxytoca* isolates. Graphs show the data from PAP assays for the six test isolates (a) *CT04*, (**b**) *Nasal2a*, (**c**) *Kleb405*, (**d**) *Blood4a*, (**e**) *ACN* and (f) *Kleb401*. For each isolate, a single colony of resistant sub-population (*CT04*^R^, *Nasal2a*^R^, *Kleb405*^R^, *Blood4a*^R^, *ACN*^R^ and *Kleb401*^R^) was re-assayed using PAP experiments to determine the stability of the phenotype. Three independent biological replicates (*N*=3) were performed, and the log of the mean±sem c.f.u.s was plotted. The detection limit for viable colonies was 100 c.f.u. ml^−1^, corresponding to log 2 on the *y*-axis.

To definitively assess whether the *K. oxytoca* isolates were heteroresistant to polymyxin B, we employed the PAP assay, which enables detection and characterization of resistant sub-populations of bacteria. Using this approach, we detected sub-populations of highly polymyxin B-resistant (MIC ≥16 µg ml^−1^) bacteria in 5/6 isolates (Fig. 2). The exception, *Kleb05*, had a sub-population that was resistant up to 8 µg ml^−1^ polymyxin B (Fig. 2).

Based on the PAP assay, we defined the MIC of the bulk population as the highest concentration of polymyxin B at which there was complete population growth (i.e. the number of c.f.u. was the same as for zero antibiotic). For all strains, this was 1 or 2 µg ml^−1^ polymyxin B, which is considered susceptible [26] (Fig. 2). Since there was detectable growth of polymyxin B-resistant sub-populations at 8–16-fold higher concentrations than the MIC of the bulk population, this meets the definition of heteroresistance [21] (Fig. 2). However, the size of the sub-population varied between strains from ~10^−6^ (*Kleb401*, *Nasal2a*, *CT04*, *Blood4a*, *ACN*) to only 10^−7^ for *Kleb05*. This explains why we observed skipped wells for most strains, since there was a ~50% chance of at least one resistant cell being present in each well of the broth microdilution assay, but not for Kleb05, where there was only a ~5% chance of a resistant cell in each well (Fig. 1).

To determine the stability of the resistant sub-population, a single colony was picked from agar plates containing 32 µg ml^−1^ polymyxin B (designated: *CT04*^R^, *Nasal2a*^R^, *Kleb405*^R^, *Blood4a*^R^, *ACN*^R^ and *Kleb401*^R^), grown in antibiotic-free MHB, and then subjected to the PAP assay again. All six isolates showed high-level, homogeneous resistance, with MICs >128 µg ml^−1^, the maximum concentration used in the assay, indicative of stable resistance (Fig. 2).

For each resistant isolate, there was no significant difference between the c.f.u. counts observed at different Polymyxin B concentrations relative to the no-antibiotic control, except at 128 µg ml^−1^, where there was a reduction, as determined by the Dunnett’s multiple comparisons test (two-way ANOVA) (Fig. 2). Notably, 4/6 isolates (*ACN*^R^, *Blood4a*^R^, *Nasal2a*^R^, *CT04*^R^) showed the presence of small colonies on plates with higher PMB concentrations (64 and 128 µg ml^−1^), which may indicate that growth at these concentrations was slowed but not inhibited by the antibiotic. However, there was no significant difference in the growth profiles of susceptible and heteroresistant populations in the absence of the antibiotic (Fig. S1). Furthermore, when we picked ten polymyxin-resistant colonies from isolate ACN is the isolate identification name as stated, we found that they all had the same high-level polymyxin B resistance as determined by PAP, suggesting an otherwise homogeneously resistant sub-population (Fig. S2).

### Characterization of polymyxin B heteroresistance at log and stationary phase

**Fig. 3.**
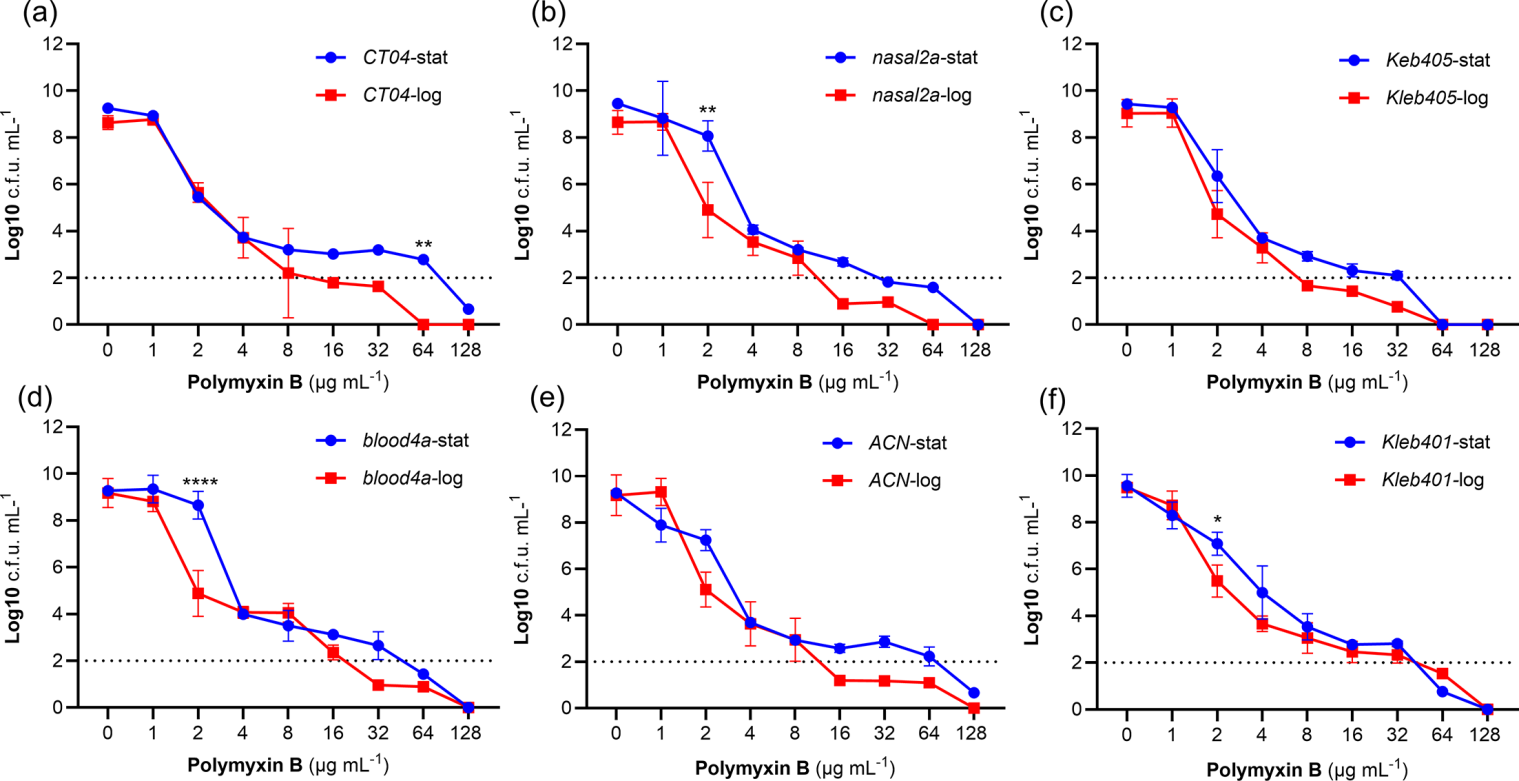
Comparison of heteroresistant sub-populations under log and stationary phase. Three independent PAP experiments were performed from log- and stationary-phase cultures. Graphs show the log of the mean±sem c.f.u.s of isolates in the presence of twofold increasing concentrations of polymyxin B. The difference between heteroresistance sub-populations under these conditions was statistically determined using two-way ANOVA (Šídák’s multiple-comparisons test), with the limit of detection at 100 c.f.u. ml^−1^ (log 2 on the *y*-axis). Significant differences between log- and stationary-phase cultures for a given concentration are indicated (*).

To understand whether the size of the resistant sub-population was influenced by growth phase, we undertook PAP experiments with log- and stationary-phase bacteria. This revealed very similarly sized resistant sub-populations in each growth phase, with only very few differences seen for strains *Blood4a*, *Nasal 2* a and *CT04* at certain antibiotic concentrations (Fig. 3). Therefore, we concluded that growth did not significantly affect the heteroresistant phenotype.

### Growth of *K. oxytoca* in human serum does not influence polymyxin heteroresistance

Polymyxin resistance is associated with resistance to host innate defences such as antimicrobial peptides (AMPs) and lysozyme [27,30]. Therefore, we hypothesized that exposure to host innate defences would select for larger polymyxin-resistant sub-populations. Since serum contains physiological concentrations of both lysozyme and AMPs, we used this to mimic the innate defences that *K. oxytoca* encounters during infection.

**Fig. 4.**
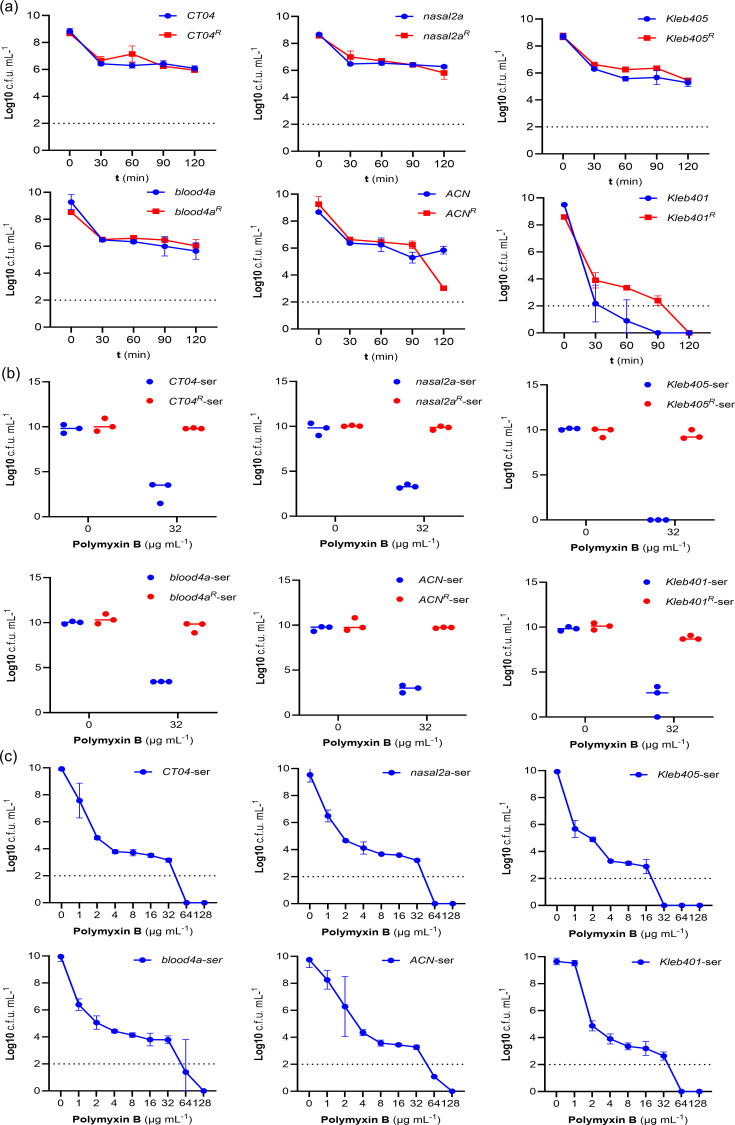
Serum resistance profile of *K. oxytoca*. (**a**) The graph represents three independent time-kill assays of susceptible (*CT04*, *Nasal2a*, *Kleb405*, *Blood4a*, *ACN* and *Kleb401*) and resistant isolates (*CT04*^R^, *Nasal2a*^R^, *Kleb405*^R^, *Blood4a*^R^, *ACN*^R^ and *Kleb401*^R^) incubated in 100% human serum for 120 min. Each time point represents the mean±sem of c.f.u. counts from the three replicates. (**b**) Polymyxin B susceptibility after growing isolates in serum-adapted MHB. Polymyxin-susceptible and resistant isolates were grown in 50% serum overnight and plated on agar containing PMB (32 µg ml^−1^). The data represent the c.f.u. counts from three independent experiments. (**c**) Serum has no effect on polymyxin heteroresistance in *K. oxytoca*; the log of the mean±sem c.f.u.s of three independent biological replicates is shown. The difference between heteroresistance sub-populations from serum-adapted and MHB-grown isolates was statistically determined using two-way ANOVA (Šídák’s multiple-comparisons test).

To do this, we first compared the survival of strains and their associated resistant isolates in 100% human serum (not heat inactivated). All the isolates except *Kleb401* survived at high levels in serum, and there was very similar survival between WT and resistant isolates (Fig. 4a). There were also very similar outcomes for WT and resistant isolates when strains were compared for growth in MHB containing human serum (Fig. S3). Furthermore, growth of isolates in MHB containing 50% human serum did not significantly affect the polymyxin susceptibility of the isolates (Fig. 4b), nor did it change the polymyxin heteroresistance phenotype in PAP assays (Fig. 4c). Combined, these data demonstrated that exposure of *K. oxytoca* to the physiologically relevant innate defences found in human serum had no impact on the polymyxin heteroresistance phenotype.

### Lipid A modifications in polymyxin B-resistant sub-populations of *K. oxytoca*

**Fig. 5.**
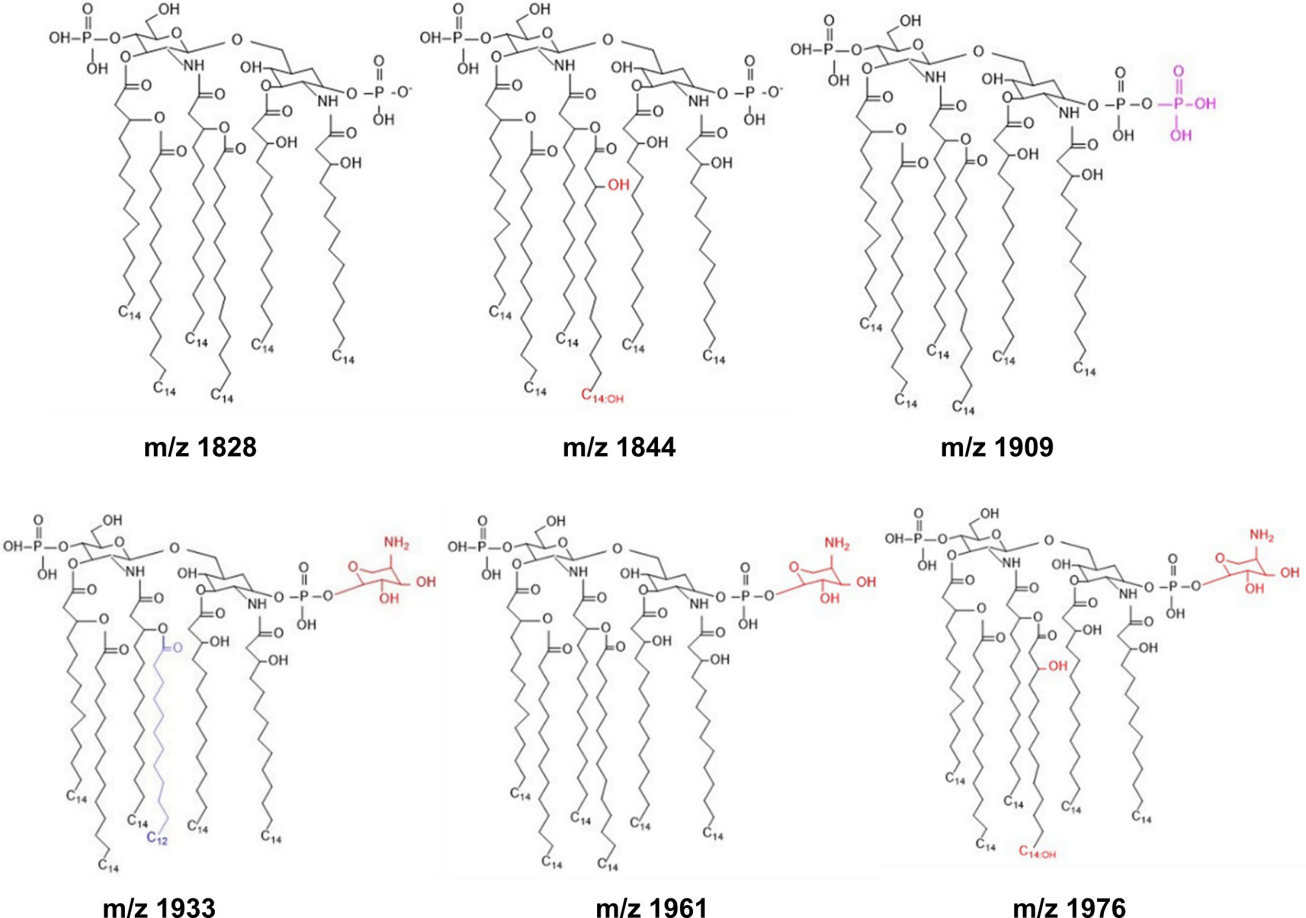
Lipid A profiling of susceptible and heteroresistant isolates, showing proposed structures of native and modified lipid A species in susceptible and resistant populations of *K. oxytoca*.

To understand the underlying mechanism of polymyxin resistance, the lipid A species present in WT and resistant isolates were profiled by mass spectrometry. Four lipid A species with mass-to-charge ratios (m/z) 1828, 1844, 1909 and 1933 were detected in the WT isolates (Fig. S4). By contrast, resistant isolates showed two additional peaks centred at m/z 1976 and m/z 1961, which indicated modification of two lipid A species (1844 and 1828) with the addition of 4-amino-l-arabinose (l-Ara4N) (m/z+131). There was no evidence of phosphoethanolamine (pEtN) modification. The proposed structure and components of the native and modified lipid A species are shown in Fig. 5. Quantification of the relative abundance of each lipid A species showed variations between WT and resistant isolates, but 2/3 species with l-Ara4N were only present in the resistant isolates, consistent with a causative role in polymyxin resistance, although additional work is needed to definitively demonstrate this (Table 1).

**Table 1.** Relative percentage abundance of native (unmodified) and modified lipid A species in susceptible and resistant sub-populations of *K. oxytoca* isolates

Strain		LPS modification	(%) Unmodified lipid A				(%) Modified lipid A	
			M/z 1828	M/z 1844	M/z 1909	M/z 1925	M/z 1961	M/z 1976
**WT**	***Kleb405***	None	46.8	2.3	48.7	2.2	0	0
	***ACN***	None	26.3	25.7	30.0	18.1	0	0
	***CT04***	None	24.4	26.9	25.3	23.3	0	0
	***Kleb401***	None	28.9	22.2	29.1	19.8	0	0
	***Blood4a***	None	24.3	19.8	32.5	23.5	0	0
	***Nasal2a***	None	22.4	20.2	31.2	26.2	0	0
**Resistant**	***Kleb405^R^***	l-Ara4n	24.1	21.0	17.0	10.1	18.8	9.0
	***ACN^R^***	l-Ara4n	14.6	16.5	3.0	3.0	29.6	33.3
	***CT04^R^***	l-Ara4n	14.1	16.5	14.5	16.7	14.6	23.6
	***Kleb401^R^***	l-Ara4n	12	35.8	10.9	16.1	9.2	16.1
	***Blood4a^R^***	l-Ara4n	14.9	19.8	11	16.9	13.6	23.9
	***Nasal2a^R^***	l-Ara4n	12.8	16.7	25.00	13.7	11.1	20.7

## Discussion

The emergence of multidrug-resistant *K. oxytoca* isolates in clinical settings is becoming a major public health threat. Importantly, heteroresistant bacteria have frequently been associated with recurrent infections, posing a major challenge to antibiotic therapy [20]. In this pilot study, *K. oxytoca* isolates from patient samples and hospital environments were found to be resistant to multiple antibiotics of different classes, including levofloxacin, chloramphenicol, imipenem and trimethoprim, with high MIC values. In previous studies, phylogenetic analysis of diverse *K. oxytoca* isolates has indicated the presence of AMR genes that confer resistance to the classes of antibiotics profiled in this study [3].

Furthermore, all six isolates profiled in this study demonstrated heteroresistance to polymyxin B, a last-resort antibiotic for treating infections caused by multi-drug resistrant (MDR) Gram-negative bacteria. Heteroresistance was evident through the observation of skipped wells in broth microdilution assays, an indication of a heterogeneous bacterial response to antibiotics [31]. The phenotype was confirmed with PAP assays, the gold standard for detecting antibiotic heteroresistance. Polymyxin heteroresistance has often been reported in *Enterobacteriales*, but with great variability in frequencies across different regions. Therefore, given the relatively small sample size used here, further analysis with a larger and more geographically diverse panel of isolates is warranted to fully understand the frequency of heteroresistance in *K. oxytoca*.

It was not surprising to find polymyxin heteroresistance at a high frequency in *K. oxytoca*, based on previous work with other *Enterobacterales* or *K. pneumoniae*. For example, a 3-year study of bloodstream isolates of the *Enterobacter cloacae* complex from healthcare centres in Germany reported the widespread prevalence of colistin heteroresistance with a frequency of 48.4% and MIC range between 64 and 512 µg ml^−1^ [32]. In a large U.S. surveillance of 408 carbapenem‐resistant *Enterobacterales* isolates collected between 2012 and 2015, colistin heteroresistance was detected in 10.1% of isolates, although 93.2% of those were misclassified as susceptible by routine tests [19]. This highlights the tendencies of the phenotype to escape clinical detection and supports the need for a more robust detection method, especially in healthcare settings. The first U.S. report of polymyxin heteroresistance in *Klebsiella* species was demonstrated in two distinct isolates of *K. pneumoniae*, where resistant sub-populations had colistin MICs up to 100 µg ml^−1^, while the main population remained susceptible at 2 µg ml^−1^ [18]. Similarly, Halaby *et al*. [33] reported colistin heteroresistance in ESBL-producing *K. pneumoniae* clinical isolates, with resistant sub-population MICs ranging from 8 to 64 µg ml^−1^ as determined by PAP assay. Subsequently, several reports of polymyxin heteroresistance in *K. pneumoniae* have emerged, with a large study from China reporting a frequency of 6.2% [34]. However, a study of 109 patients in China with carbapenem-resistant *K. pneumoniae* infection detected polymyxin heteroresistance in 70% of cases [35]. In stark contrast to these studies, analysis of 154 *K*. *pneumoniae* isolates from a tertiary care hospital in Turkey failed to identify any colistin heteroresistance [36], underscoring the variation in reported frequencies between studies. A recent meta-analysis of 18 studies of polymyxin heteroresistance in *K. pneumoniae* further exemplifies the variation in reported frequency, with an overall occurrence of 31.5%, ranging from <1% to 100% [37].

In addition to the challenge of detecting heteroresistance, the relative importance of *K. oxytoca* is often under-recognized due to the lack of routine speciating methods in clinical diagnostics in resource-poor settings [38,39]. As such, this study provides the first characterization of polymyxin heteroresistance in this species.

We observed similar heteroresistance phenotypes in *K. oxytoca* to those reported for *K. pneumoniae*, with high MICs of the resistant sub-populations ranging from 32 to 128 µg ml^−1^. The emergence of heteroresistance in these *K. oxytoca* isolates suggests an ability to cause persistent or recurrent infections, as indicated in *in vivo* experiments with heteroresistant *K. pneumoniae* isolates [18]. The isolates profiled in this study showed a heteroresistance phenotype such that a single colony of each isolate produces a small, highly resistant sub-population, the size of which was not affected by bacterial or host environmental factors such as log phase, stationary phase and human serum.

Polymyxin B is a membrane-targeting cationic antibiotic that primarily interacts with the negatively charged LPS of the bacterial membrane [40,40]. Polymyxin resistance has been associated with structural modification of the LPS, such as the addition of l-Ara4N or pEtN to the lipid A component [25]. These modifications have also been linked to colistin resistance and heteroresistance in *K. pneumoniae* and *E. cloacae,* respectively [41]. Hence, we profiled the lipid A of all six *K. oxytoca* isolates to identify modifications that may be implicated in the observed phenotype. Four native lipid A species were identified in the susceptible population of the six isolates. In the resistant sub-populations, however, these native lipid A species were modified by the addition of an l-Ara4N moiety, consistent with previous observations in *K. pneumoniae*.

*K. oxytoca* is an emerging threat, particularly in healthcare settings, due to high rates of antibiotic resistance [15,39]. In low- and middle-income countries, where access to new antibiotics is very limited, polymyxin is increasingly used to treat infections caused by multidrug-resistant Gram-negative pathogens [10,12]. Whilst the full significance of heteroresistance is not yet understood, at least in part because it is hard to detect, there is a growing body of evidence to suggest that it can contribute to treatment failure, including animal and clinical studies, resulting in persistent or relapsing infection [18,20, 42]. As such, polymyxin heteroresistance may very likely further limit treatment options for infections caused by *K. oxytoca* and similar pathogens.

In summary, this study is the first to characterize polymyxin heteroresistance in *K. oxytoca*, as well as factors that could potentially influence the phenotype. These isolates each produce a sub-population of stably polymyxin-resistant bacteria, the size of which is not influenced by bacterial growth phase or innate host immune factors. As an emerging threat in healthcare settings, we suggest further studies to explore the mechanisms driving polymyxin heteroresistance to inform appropriate diagnostic and therapeutic strategies, including combinations of antibiotics available to those in low-income settings [43,44].

## Supplementary material

10.1099/jmm.0.002148 Uncited Supplementary Material 1.

## Abbreviations

AMPs: antimicrobial peptides
ECC: *Enterobacter cloacae* complex
ESBL: extended-spectrum beta-lactamase
ICU: intensive care unit
l-Ara4N: 4-amino-l-arabinose
MALDI-TOF: matrix assisted laser desorption/ionization-time of flight
*mcr*: mobile colistin resistance
PAP: population analysis profiling
pEtN: phosphoethanolamine
