# Supplementary material for: Polymyxin heteroresistance in Klebsiella oxytoca
Source: J Med Microbiol. 2026 Mar 27;75(3):002148. doi: 10.1099/jmm.0.002148 (PMC13030853; doi:10.1099/jmm.0.002148)
Supplement: Uncited Supplementary Material 1. [file jmm-75-02148-s001.pdf]

Supplementary Data

Table S1: Antimicrobial susceptibility profiles of *K. oxytoca* isolates

**Antibiotics**	**Minimum inhibitory concentration (µg/mL)** **Bacterial strains**					
	Kleb401	***Nasal2A***	***CT04***	***Blood4a***	***ACN***	***Kleb405***
**Ceftazidime**	0.25	2	2	2	1	0.5
**Levofloxacin**	64	64	64	64	32	32*
**Chloramphenicol**	>64	>64	>64	>64	>64	64
**Colistin**	0.5/16*	2/2	8/32*	64/64*	8/32*	0.25/8*
**Polymyxin B**	2/16*	8/32*	2/16*	32/32	2/16*	0.25/0.25
**Gentamicin**	0.25	0.25	0.25	0.25	0.2	0.25
**Imipenem**	16	16	32	32	32	16
**Meropenem**	0.125	0.125	0.125	0.125	0.125	0.125
**Trimethoprim**	>64	>64	>32	>64	>64	32*

For polymyxin B and Colistin, where several strains showed a skipped well phenotype, we provide the lowest concentration of antibiotic that inhibited growth in at least one assay (blue), which is indicative of the MIC of the bulk population, as well as the lowest concentration at which bacterial growth never occurred (black).

*indicates skipped wells

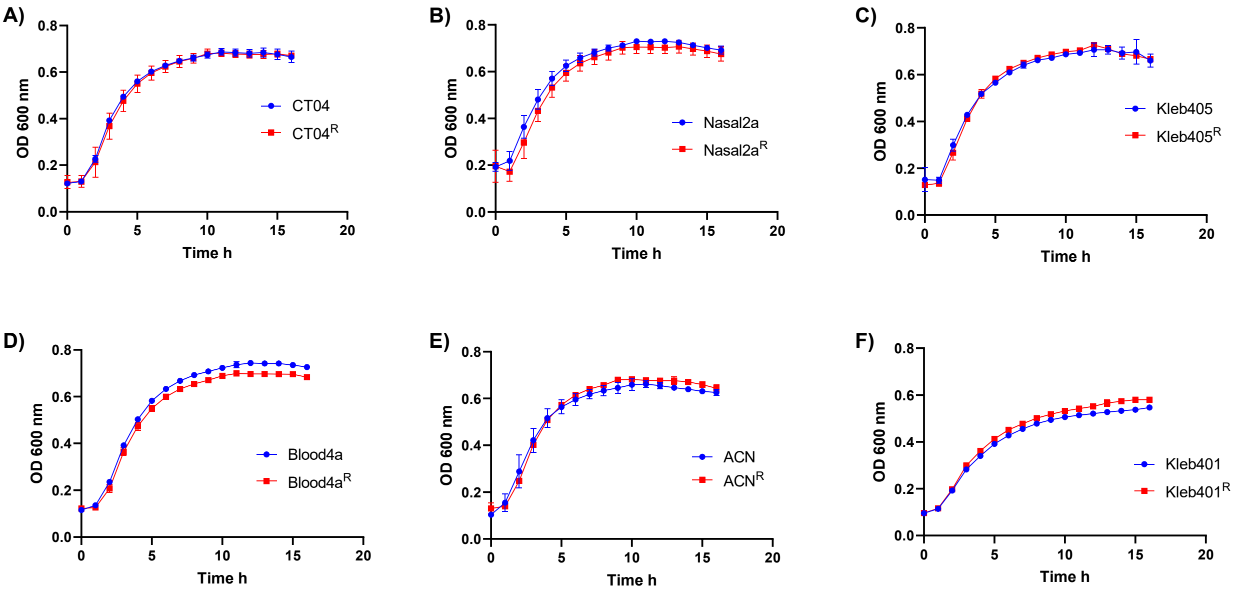

Supplementary figure S1: Growth profiles of susceptible and resistant sub-populations of all six isolates. The mean±SEM OD_{600} values from three independent biological replicates is shown.

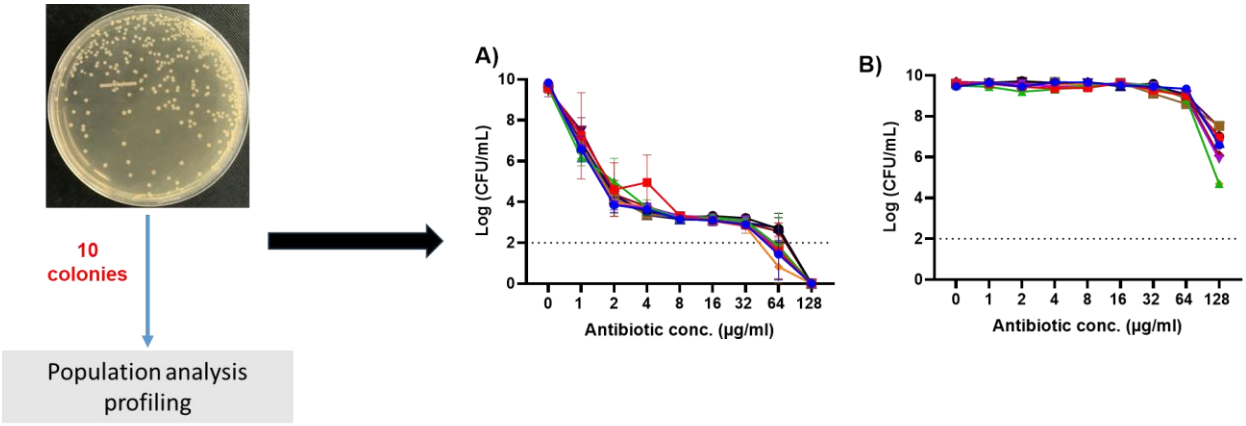

Supplementary figure S2: Determination of the stability of heteroresistance in *K. oxytoca (ACN)*; ten colonies of the isolate (ACN) were randomly selected from MH agar plate and subjected to PAP experiment **(A)**. Heteroresistant isolates obtained from MH agar containing 32 µg/mL of polymyxin B were taken through another round of PAP experiment **(B)**. The graphs show the mean±SEM of three independent experiments.

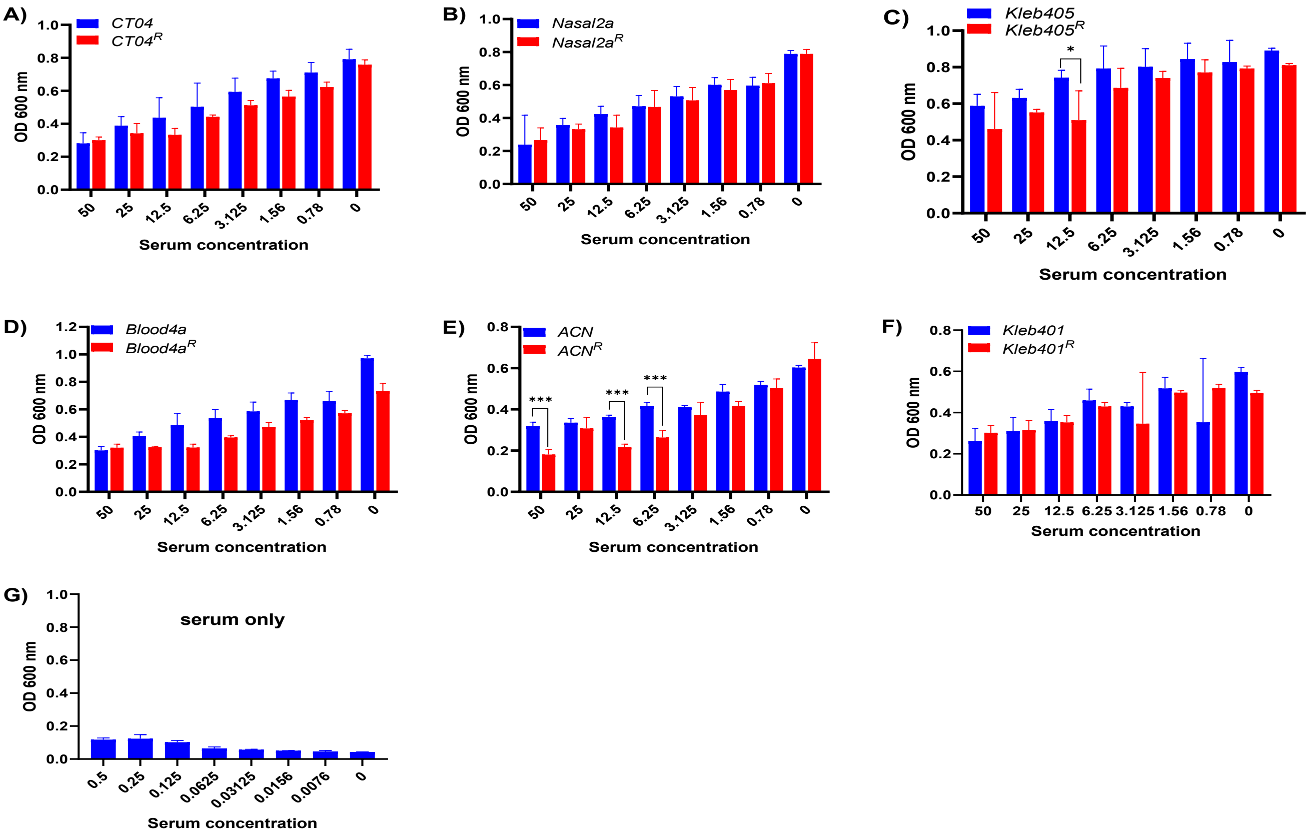

Supplementary figure S3: Serum growth assay. Growth of susceptible and resistant isolates in MHB containing various concentrations of serum. The graphs show the mean±SEM of OD_{600} measurement from three independent experiments. Statistical difference between bacterial survivals at different serum concentrations compared to the control was determined using two-way ANOVA (Šídák's multiple comparisons test).

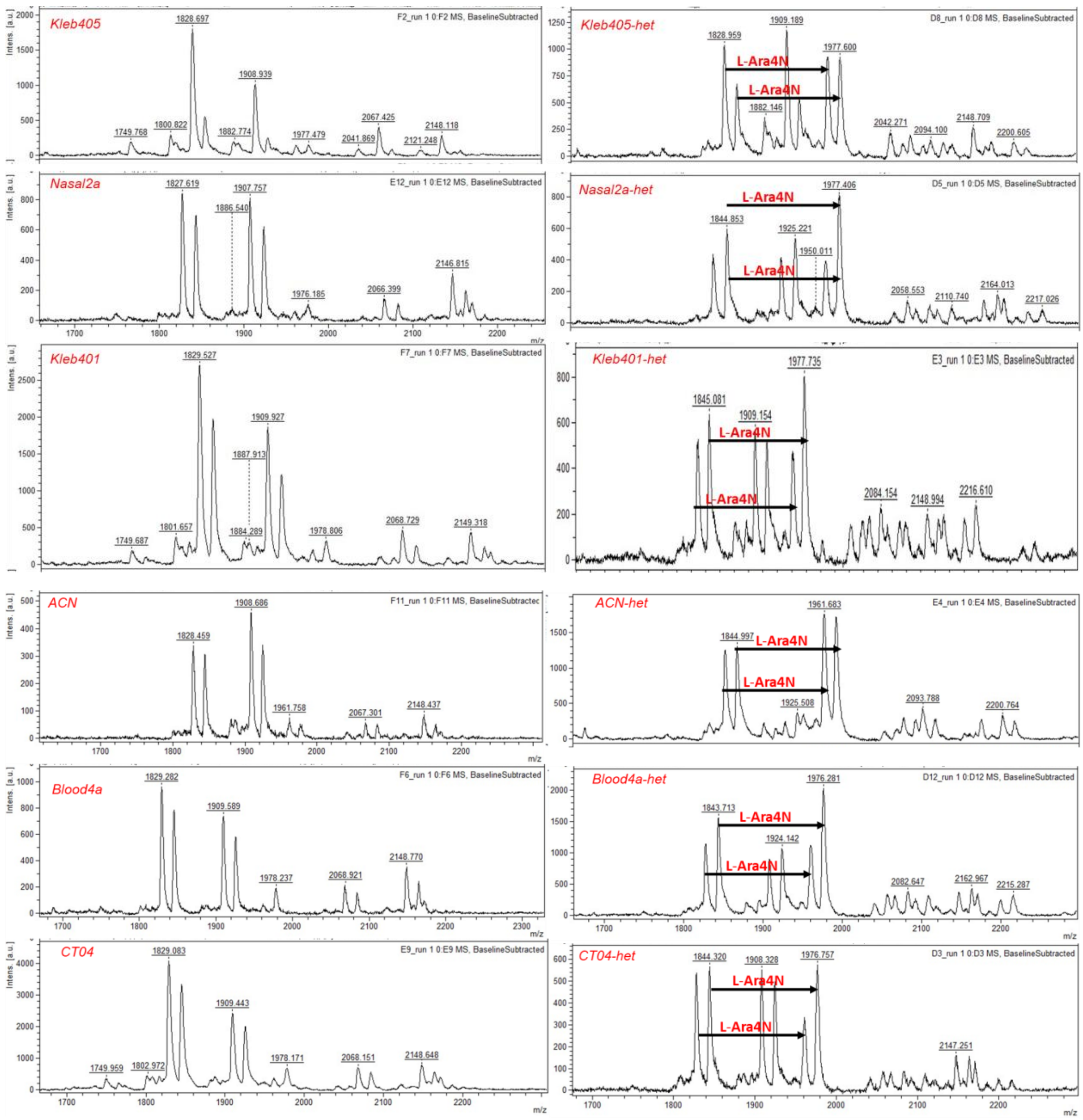

S.4: Representative mass spectra of polymyxin susceptible and resistant sub-populations of all six isolates *(Kleb405, Nasal2a, Kleb401, ACN, Blood4a, and CT04).*
